# The human lung microbiome—A hidden link between microbes and human health and diseases

**DOI:** 10.1002/imt2.33

**Published:** 2022-06-16

**Authors:** Xinzhu Yi, Jingyuan Gao, Zhang Wang

**Affiliations:** ^1^ Institute of Ecological Sciences, School of Life Sciences South China Normal University Guangzhou Guangdong China

**Keywords:** gut–lung axis, lung microbiome, microbiome–host interaction, respiratory diseases

## Abstract

Once thought to be sterile, the human lung is now well recognized to harbor a consortium of microorganisms collectively known as the lung microbiome. The lung microbiome is altered in an array of lung diseases, including chronic lung diseases such as chronic obstructive pulmonary disease, asthma, and bronchiectasis, acute lung diseases caused by pneumonia, sepsis, and COVID‐19, and other lung complications such as those related to lung transplantation, lung cancer, and human immunodeficiency virus. The effects of lung microbiome in modulating host immunity and inflammation in the lung and distal organs are being elucidated. However, the precise mechanism by which members of microbiota produce structural ligands that interact with host genes and pathways remains largely uncharacterized. Multiple unique challenges, both technically and biologically, exist in the field of lung microbiome, necessitating the development of tailored experimental and analytical approaches to overcome the bottlenecks. In this review, we first provide an overview of the principles and methodologies in studying the lung microbiome. We next review current knowledge of the roles of lung microbiome in human diseases, highlighting mechanistic insights. We finally discuss critical challenges in the field and share our thoughts on broad topics for future investigation.

## Highlights

- Sputum, bronchoalveolar lavage, bronchial brushing, and lung tissue are the routine sample types for the lung microbiome. Multiomics have been increasingly applied for characterizing the lung microbiome.
- Lung microbiome is broadly implicated in chronic and acute lung diseases, lung cancer, and other lung diseases, with potential mechanistic implications.
- Current challenges of the lung microbiome studies include low microbial‐to‐host ratio, high disease heterogeneity, and the difficulty in precisely manipulating and culturing the lung microbiome.
- Future potential topics for lung microbiome studies include understanding the diagnostic potential of the lung microbiome, its spatial dynamics, its mechanistic interaction with the host, and the crosstalk between the lung and distal organs.

## Graphical abstract

Broad topics of the human lung microbiome are reviewed, including its principles and methodologies, applications to human diseases, mechanistic insights, current challenges, and future potential research avenues.

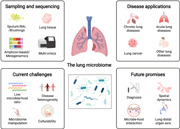

## INTRODUCTION

As the “second genome” of the human body, the human microbiome plays a crucial role in human health and diseases, and has received extensive attention over the past decades [1]. Compared to the topic of gut microbiota, which has dominated the human microbiome studies, much less attention has been paid to the microbiome of the human respiratory tract, partly due to historical consideration of the healthy lung as a sterile organ over a century. The dogma of lung sterility has been overturned with the advent of culture‐independent sequencing techniques that led to the first discovery of a microbial community in the airway by Hilty et al. [2]. The field of lung microbiome has since witnessed exponential growth. Compelling evidence from human studies has demonstrated that the lung microbiome is altered in a broad range of lung diseases, such as chronic lung diseases (i.e., asthma, chronic obstructive pulmonary disease [COPD], bronchiectasis, and idiopathic pulmonary fibrosis [IPF]), acute lung diseases (i.e., pneumonia, sepsis, acute respiratory distress syndrome [ARDS], and COVID‐19), and complications postlung transplantation, human immunodeficiency virus (HIV), tuberculosis, and lung cancer. Emerging animal studies have further revealed a mechanistic implication of the lung microbiome in regulating host pathophysiological processes both locally and distally, together uncovering a hidden link between the lung microbiome and human diseases. Nevertheless, compared to the rapid advancement of gut microbiome studies, the field of lung microbiome is still in its infancy and facing a series of critical challenges stemming from the unique anatomy of the lung and the microbial biomass in the lung that is orders of magnitude lower than that in the gut, necessitating the development of novel approaches tailored for the lung microbiome. Here, we review the broad topic of the human lung or lower respiratory tract microbiome, including its principles and methodologies, applications to human diseases, current challenges, and future potential research avenues, in the hope that this review will serve as a catalyst to stimulate greater interest in the burgeoning field of human lung microbiome.

## METHODOLOGIES ON THE LUNG MICROBIOME

### Sampling the lung microbiome

**Figure 1:**
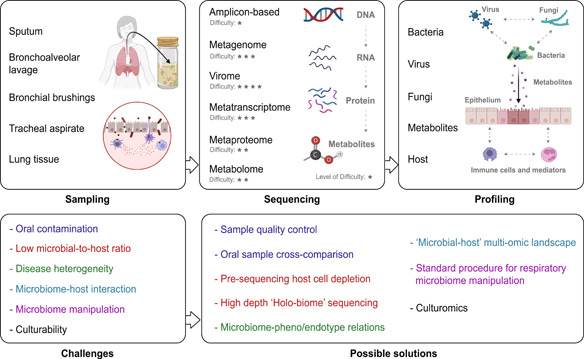
The principles and methodologies of studying the human lung microbiome, including sampling approaches, sequencing strategies, and the microbiome and host profiles that can be obtained. For each type of sequencing, the level of difficulty is scored based on the empirical assessment of technical challenges. The challenges in the field of the lung microbiome as well as possible solutions to address each of them are also shown.

Despite sharing most principles established for the gut microbiome in terms of sequencing and data analyses, the lung microbiome has its unique aspects in methodologies particularly with respect to sampling (Figure 1). In essence, it is impractical to directly obtain the human lung tissue unless surgically justified (i.e., lung transplantation, tumor resection). As such, several noninvasive and invasive procedures have been implemented as a surrogate or proxy to sampling the lung environment. Of them, sputum has been one of the most commonly used specimens for studying the airway microbiota, due to its noninvasive nature, which facilitates sample collection particularly for patients with chronic lung diseases who are often able to produce sputum spontaneously. For patients or healthy individuals who are unable to do so, sputum induction using nebulized saline is a routine procedure that is clinically safe and effective [3]. Therefore, sputum remains the most viable option to study the airway microbiome for healthy individuals. However, the extent of sputum samples in representing the lower airways is the subject of debate, given its inherent admixture of materials from upper, lower airways and the oral cavity [4]. As such, a process for separating sputum plugs (the mucous part of a sputum) from saliva, followed by a quality assessment (i.e., via microscopy inspection of the leukocyte/squamous epithelial cell ratio), should be conducted for sputum samples to minimize oral contamination [5]. In addition, the concurrent oral rinse sample from the same individual can be used as a control to assess oral contamination [6].

Bronchoalveolar lavage (BAL) is another frequently used approach to sample the lung microbiome. Operated via bronchoscopy, BAL is invasive and more costly and time‐consuming than sputum sampling, posing a challenge for longitudinal sample collection. However, the clear advantage of BAL over sputum lies in its better resemblance to lower airways, with limited upper airway or oral contamination. Other approaches in sampling the lung microbiome include bronchial brushing and tracheal aspirate, which has been applied on a limited basis [7, 8, 9, 10]. Theoretically, lung tissue is the most ideal specimen to study the lung microbiome and has the unique merit in capturing the topographical distribution of microbial communities [11]. However, the inability to obtain lung tissue in most clinical conditions has limited its application only to patients receiving lung resection, cancer‐related surgery, or biopsy.

### Sequencing the lung microbiome

The substantially low microbial biomass in the respiratory tract compared to that in gut and oral cavity calls for special attention to sampling, processing, and analysis of the lung microbiome (Figure 1). For sample processing, bacterial genomic DNA is present in reagents used for DNA extraction and polymerase chain reaction (PCR) [12]. This impact of reagents will further magnify when the concentration of the source DNA is low. Therefore, while negative reagent controls have often been neglected for gut microbiome studies, it is a standard practice for all lung microbiome studies to include negative reagent controls, in which nuclease‐free water is used in place of the real samples throughout DNA extraction, PCR, and sequencing [13]. The identified bacterial taxa in the reagent controls are often removed from the real samples in downstream analyses. Alternatively, they can be explicitly flagged and reported as contaminants and be retained in the real data, as simply excluding them could also remove potentially “true” signals and bias the overall observation, given the compositional nature of microbiome data [14].

For sequencing strategies, 16S ribosomal RNA (rRNA) gene‐based amplicon sequencing is widely applied to lung microbiota studies due to its technical ease and robustness. The V4 hypervariable region of the 16S rRNA gene is the most frequent choice of sequencing, which is, however, incapable of providing in‐depth taxonomic resolution (i.e., mostly up to the genus level) [15]. Powered by third‐generation long‐read sequencing (i.e., PacBio), recent studies have characterized the species or even the strain level of the lung microbiome by sequencing the full‐length 16S rRNA gene, uncovering additional microbial diversity and heterogeneity [16, 17]. It is known that 16S rRNA gene sequencing has inherent biases, largely ascribed to the differential efficiency of PCR amplification of the 16S rRNA gene from individual bacterium [18]. The copy number variation of the 16S rRNA gene among bacterial species further leads to biased cell abundance estimation [19]. Metagenomic sequencing has demonstrated its strength in profiling the functional capacity of the microbiome, moving the scientific focus from “who is there” to “what can they do” [20]. The metagenomic approach is generally considered amplification‐free. However, whole‐genomic amplification may occasionally be applied to low‐biomass samples to increase the DNA quantity, which can introduce additional biases [21]. However, its application to the lung microbiome remains largely scarce, hindered by an intimidatingly high host‐to‐microbial DNA ratio in the lung compartments. As a result, the vast majority of metagenomic sequencing reads will come from the host. Certain methods and commercial kits have been developed to deplete host genomic DNA before sequencing [22, 23], which, however, have shown varied efficiency with a critical risk of concomitantly removing bacterial DNA. Sequencing the host‐microbial “holo‐biome” and filtering the host reads postsequencing represent a viable approach [24], and yet, the high sequencing depth required to achieve sufficient microbial coverage makes this approach cost‐inhibitive. The same limitation also applies to other amplification‐free sequencing approaches such as metatranscriptomics.

Compared to the bacterial microbiome, the fungal and viral components of the lung microbiome, despite their critical importance, remain largely unexplored until recently (Figure 1). The lung fungal microbiome (or mycobiome) can be characterized by sequencing the 18S rRNA gene or the internal transcribed spacer (ITS) region. Extraction of fungal DNA requires additional procedures (i.e., bead‐beating) to break the fungal cell wall [25]. The incomplete fungal taxonomic reference database is a technical bottleneck for airway mycobiome studies, resulting in suboptimal fungal taxonomic assignment [26]. The variable length of the ITS region across fungal species further complicates the procedure for sequencing reads processing and taxonomic identification [27]. Although respiratory pathogenic viruses are well characterized clinically (i.e., by multiplex quantitative PCR) [28], the overall viral community or virome in the lung remains poorly understood, largely due to the methodological challenges for virome sequencing. A purification and enrichment step is required to isolate the viral particles and eliminate nonviral components before viral DNA/RNA extraction. Implementation of this approach in airway samples is challenging, however, due to the unique features of sample types (i.e., high viscosity), and the low abundance and fragility of viral components. It is noteworthy that a recent study has shown the feasibility in characterizing the sputum virome, revealing a much stronger association between the virome and clinical parameters in asthma compared to bacteriome [29]. Finally, although largely understudied, archaea were also found to harbor the human lung, with members of *Woesearchaeota* (DPANN superphylum) identified as the dominating lung archaeal taxon [30].

The human microbiome studies have entered a multiomics era [31]. Integration of multiple omics along the microbiome–host axis, such as metagenomics, metatranscriptomics, metabolomics, and metaproteomics, allows researchers to gain a more comprehensive insight into the functions of microbiome and its interactions with host (Figure 1). While multiomics are increasingly being applied to gut microbiome studies [32, 33], its implementation in the lung microbiome remains sparse. Untargeted metabolomics characterization is routinely applied to airway samples (i.e., sputum, BAL) for exploratory and hypothesis‐generating purposes [34, 35]. The levels of key metabolites of interest are often validated using targeted metabolomics with a reference standard. Metaproteomics are a promising technique and increasingly being used to gain unique insights into microbiome–host interactions by characterizing functional proteins from specific microbial taxa and host [36]. However, due to its relatively high cost, the application of metaproteomics to respiratory studies remains scant [37, 38].

## THE LUNG MICROBIOME IN HUMAN HEALTH AND DISEASES

### The healthy lung microbiome

Due to the unique topographic structure of the lung, which is constantly exposed to the environment, the lung microbiome is in an ecologically dynamic state, inherently shaped by three factors: microbial immigration (i.e., via microaspiration from the upper respiratory tract), microbial emigration or clearance, and replication of the local microbes [39]. Firmicutes and Bacteroidetes are the predominant phyla in healthy lung microbiota, with *Prevotella*, *Veillonella*, and *Streptococcus* being the most common genera [11, 40]. In healthy individuals, the lung microbiome composition is determined by a balance between microbial immigration and emigration, with limited contribution from local microbial replication [41]. In the disease state, the alterations in the lung structure and the local microenvironment, including mucosa pH, oxygen gradient, nutrient availability, temperature, and inflammation, promote microbial growth, leading to an altered composition of lung microbiota (i.e., increased Proteobacteria).

**Figure 2:**
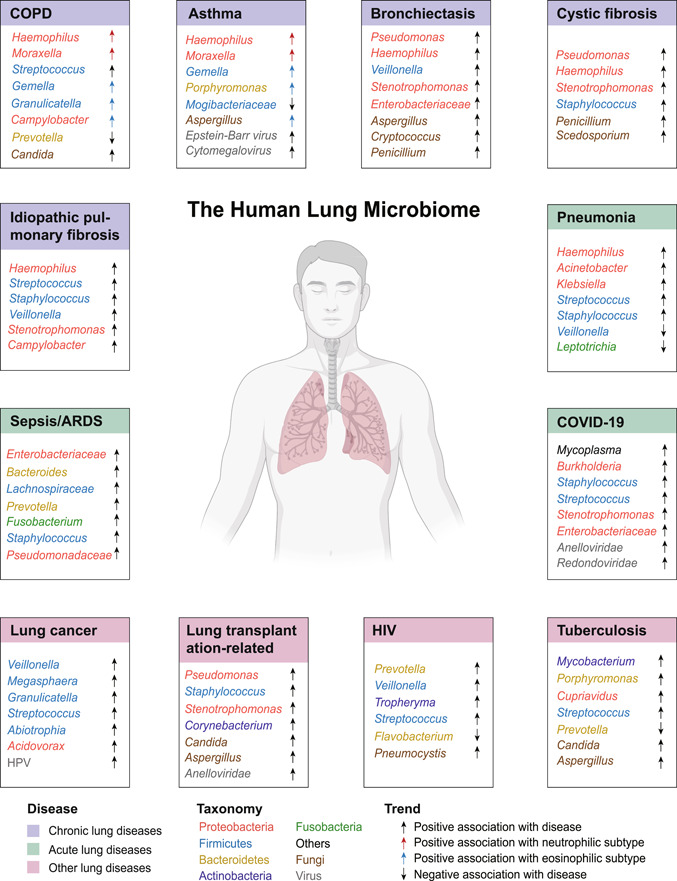
Applications of the human lung microbiome to disease areas, categorized by chronic lung diseases, acute lung diseases, and other lung diseases. For each disease, the bacteria, viral, or fungi taxa positively (either enriched in disease vs. controls or positively associated with key clinical features such as exacerbation, inflammation, or mortality) or negatively (either depleted in disease vs. controls or negatively associated with key clinical features) are indicated by arrows pointing upward or downward, respectively. For COPD and asthma, bacteria taxa associated with a specific inflammatory endotype, namely, neutrophilic or eosinophilic inflammation, are indicated by red or blue arrows, respectively. ARDS, acute respiratory distress syndrome; COPD, chronic obstructive pulmonary disease.

Following the above‐mentioned principles and methodologies, numerous studies have characterized the lung microbiome in human health and diseases, in which a shift of the microbiome is found in association with diseases and key clinical parameters such as severity, exacerbation, phenotype, endotype, inflammation, and mortality. This section reviews current knowledge of the lung microbiome in human diseases, spanning across chronic, acute, and other types of lung diseases (Figure 2, Table 1).

**Table 1:** Summary of key studies on the lung microbiota in chronic, acute, and other types of lung diseases

Disease	Study	Specimen	Design and sample size	Methods	Key findings
COPD	Wang et al. (2016)	Sputum	Stable, exacerbations, posttherapy, recovery, 476 samples, 87 patients	16S V3–V5	● *Moraxella* increased in exacerbations. ● Proteobacteria increased in bacterial versus eosinophil exacerbations.
COPD	Wang et al. (2018)	Sputum	Stable, exacerbations, 715 samples, 287 patients	16S V4	● *Veillonella* decreased in exacerbations. ● Proteobacteria increased in bacterial versus eosinophil exacerbations. ● Microbiome temporal variability increased in frequent exacerbators.
COPD	Dicker et al. (2020)	Sputum	Stable, exacerbations, 252 patients	16S V4	● Proteobacteria increased in noneosinophilic phenotype, and associated with increased mortality and neutrophil markers.
COPD	Wang et al. (2020)	Sputum	1666 Microbiome samples, 1340 human transcriptome samples	16S, metagenomics, host transcriptome	● *Haemophilius*, *Moraxella*, *Streptococcus*, and *Lactobacillus* increased in COPD. ● Butyrate, homocysteine, and palmitate associated with COPD host genes.
COPD	Wang et al. (2021)	Sputum	Stable, exacerbations, 1706 sputum samples, 510 patients	16S V4, V3–V5	● Two microbial communities in neutrophilic COPD differentiated by *Haemophilus* and associated with IL‐1β and TNF‐α, or IL‐17A. ● *Campylobacter* and *Granulicatella* were associated with sputum eosinophilia over time.
Asthma	Huang et al. (2015)	Bronchial brushing	40 Patients	16S phylochip, host transcriptomics	● Proteobacteria associated with airway leukocytes. ● Actinobacteria associated with FKBP5, an indicator of steroid responsiveness.
Asthma	Taylor et al. (2018)	Sputum	167 Patients	16S V1–V3	● *Haemophilus* was dominant in neutrophilic asthma. ● *Gemella* and *Porphyromonas* enriched in eosinophilic asthma.
Asthma	Abdel‐Aziz et al. (2020)	Sputum	100 Severe and 24 mild‐moderate asthma	16S, metagenomics	● Two microbiome clusters identified. ● The cluster 2 patients had dysbiosis, increased neutrophils, and worse outcomes.
Asthma	Sharma et al. (2019)	BAL, endobronchial brush	39 Asthma and 19 controls	16S V4, ITS	● *Fusarium*, *Cladosporium*, and *Aspergillus* enriched in T2‐high asthma. ● *Aspergillus*, *Fusarium*, and *Penicillium* correlated with FEV1.
Bronchiectasis	Guan et al. (2018)	Sputum	106 Patients and 17 controls	16S V4	● Patients divided into three groups (PA, PPM, and commensal). ● α‐Diversity decreased in the PA group. ● *Haemophilus* increased in the PPM group.
Bronchiectasis	Mac Aogain et al. (2018)	Sputum	238 Patients and 10 controls	ITS	● *Aspergillus*, *Cryptococcus*, and *Clavispora* are bronchiectasis‐associated. ● *Aspergillus terreus* associated with exacerbations.
Bronchiectasis	Mac Aogain et al. (2021)	Sputum	217 Patients and 30 controls. A validation cohort with 166 patients	16S V3–V4, ITS, viral qPCR, metagenomics	● Complexity of microbial co‐occurrence networks decreased in frequent exacerbators. ● Microbial antagonism during exacerbations, which resolves following treatment.
Cystic fibrosis	Zemanick et al. (2017)	BAL	146 Patients and 45 controls	16S V1–V2	● *Streptococcus*, *Prevotella*, and *Veillonella* associated with airway inflammation. ● *Streptococcus* predominated in patients aged <2 years.
IPF	Molyneaux et al. (2017)	BALF, blood	60 Patients and 20 controls, patients sampled longitudinally	16S V3–V5, human transcriptome	● Two gene modules are associated with airway microbiome, bacterial burden, and neutrophilia.
Pneumonia	Kitsios et al. (2018)	Endotracheal aspirates	56 Patients	16S V4	● Sequencing is congruent and more sensitive than culture in detecting pathogenic bacteria.
Sepsis/ARDS	Dickson et al. (2016)	BALF	100 Samples from 68 patients	16S V4	● Lung communities were dominated by gut‐associated bacteria (*Bacteroides* spp.). ● Alveolar TNF‐α was correlated with altered lung microbiota.
ARDS	Kyo et al. (2019)	BALF	40 Patients, 7 controls	16S V5–V6	● Microbiota diversity decreased and bacterial loads increased in ARDS. ● *Staphylococcus*, *Streptococcus*, and *Enterobacteriaceae* associated with mortality.
ARDS	Panzer et al. (2018)	Endotracheal aspirate	74 On ICU admission, 30 at 48 h after admission	16S V4	● *Streptococcus*, *Fusobacterium*, *Prevotella*, and *Treponema* associated with smoking. ● ARDS associated with *Enterobacteriaceae*, *Prevotella*, and *Fusobacterium*.
COVID‐19	Sulaiman et al. (2021)	Bronchoscopies	142 Patients	Metagenomics, metatranscriptomics, host transcriptome	● Poor clinical outcome was associated with *Mycoplasma salivarium*. ● Increased SARS‐CoV‐2 abundance and host transcriptome profile predict mortality.
COVID‐19	Zhong et al. (2021)	Sputum, nasal and throat swab, anal swab and feces	8 Mildly and 15 severely ill patients, 47 airway samples, 20 anal swab and feces	Metatranscriptomics	● *Burkholderia cepacia*, *Staphylococcus epidermidis*, or *Mycoplasma* increased in severely ill patients.
COVID‐19	Ren et al. (2021)	Oropharyngeal swab	588 Samples, 192 patients and 95 controls	Metatranscriptomics	● *Streptococcus* increased in recovered patients. ● *Streptococcus parasanguinis* correlated with prognosis in nonsevere patients.
Lung cancer	Tsay et al. (2018)	Airway brushings	39 Lung cancer, 36 noncancer, and 10 healthy controls	16S V4, human transcriptome	● *Streptococcus* and *Veillonella* increased in lung cancer. ● *Veillonella*, *Prevotella*, and *Streptococcus* associated with ERK and PI3K signaling.
Lung cancer	Tsay et al. (2021)	Bronchoscope, airway brushing	83 Lung cancer patients	16S V4, human transcriptome	● *Veilonella parvula* led to decreased survival, increased tumor burden, IL‐17 inflammatory, and activation of checkpoint inhibitor markers.
Lung transplantation	Combs et al. (2021)	BALF	134 Patients	16S, droplet digital PCR	● Increased bacterial burden, but no individual taxa associated with CLAD.
Lung transplantation	Bernasconi et al. (2016)	BALF	203 Samples from 112 patients postlung transplantation	16S V1–V2	● Lung microbiota profile aligned with distinct innate cell gene expression. ● “Inflammatory” and “remodeling” profiles linked to bacterial dysbiosis.
Lung transplantation	Watzenboeck et al. (2022)	BALF	78 Lung donors and recipients	16S V1–V2, lipidomics, and metabolome	● Recipient‐specific and environmental factors shape the long‐term lung microbiome. ● Multiomics predict future changes of FEV_1_.
HIV	Lozupone et al. (2013)	BALF	82 HIV‐positive, 77 HIV‐negative	16S, metagenomics	● *Tropheryma whipplei* increased in HIV and decreased with antiretroviral therapy.
HIV	Twigg 3rd et al. (2016)	BALF	30 HIV‐positive, 22 HIV‐negative	16S V1–V3	● *Streptococcus* increased in HIV and Flavobacterium increased in controls. ● *Prevotella* and *Veillonella* increased after 1 year of treatment.
Tuberculosis	Hu et al. (2020)	BALF	30 MTB+, 30 MTB−	16S V3–V4, metagenomics	● *Mycobacterium tuberculosis* dominated in MTB+ subjects and impacted the microbial functional profile and fungal community.
Tuberculosis	Zhou et al. (2015)	BALF	32 MTB+, 24 MTB−	16S V3	● *Cupriavidus* increased in TB. ● *Mycobacteria* and *Porphyromonas* increased inside TB lesions.

Abbreviations: ARDS, acute respiratory distress syndrome; BAL, bronchoalveolar lavage; COPD, chronic obstructive pulmonary disease; ERK, extracellular signal‐regulated kinase; HIV, human immunodeficiency virus; IL, interleukin; IPF, idiopathic pulmonary fibrosis; ITS, internal transcribed spacer; PA, pseudomonas aeruginosa; PI3K, phosphatidylinositol 3‐kinase; PPM, potentially pathogenic microorganisms; qPCR, quantitative polymerase chain reaction; SARS‐CoV‐2, severe acute respiratory syndrome coronavirus 2; TB, tuberculosis; TNF‐α, tumor necrosis factor alpha.

### Chronic lung diseases

One disease area that lung microbiome studies have extensively focused on is chronic respiratory diseases, including COPD, asthma, bronchiectasis, cystic fibrosis, interstitial pulmonary fibrosis, and so on. A key manifestation of these disorders is the chronic airway inflammation that persists throughout disease progression. In a hallmark study by Hilty et al. [2] that challenged the concept of lung sterility, the airway microbiota was found to differ between patients with COPD, asthma, and healthy controls, with elevated Proteobacteria in disease states. This pattern was subsequently supported by numerous studies demonstrating the association of members of Proteobacteria such as *Haemophilus*, *Moraxella*, and *Pseudomonas* with diseases and key clinical features. In our previous study of 476 sputum samples collected longitudinally from 87 COPD patients across stable state, exacerbations, 2‐week posttherapy, and 6‐week recovery, Proteobacteria and specifically *Moraxella* were found to be elevated in exacerbations, which was reversed posttreatment [42]. *Haemophilus* was identified as the hub node in the microbiome network and positively correlated with sputum interleukin‐8 (IL‐8) [42]. These results were further supported by our subsequent larger COPD microbiome studies on 775 sputum samples collected over 2 years from 287 COPD patients across three centers in United Kingdom [43]. The elevation of Proteobacteria was also associated with increased long‐term mortality and resistance to antimicrobial therapy for COPD patients [44, 45]. In our recent large‐scale microbiome meta‐analysis using 1666 public samples, enrichment of *Haemophilus*, *Streptococcus*, *Moraxella*, and *Lactobacillus* was found in COPD versus healthy controls [46]. In our pilot COPD multiomic study, *Haemophilus* and *Moraxella* were associated with different components of host immune and inflammatory patterns, implying their differential roles in the pathogenesis of COPD [47]. Increased Proteobacteria was also observed in asthmatic patients with the elevation of non‐Proteobacteria taxa such as *Porphyromonas*, *Fusobacterium*, and Sphingomonadaceae [48], and associated with worse clinical outcome of severe asthma [49], as well as expression of human Th17‐related genes [50]. As the key pathogenic agent, *Pseudomonas* was markedly elevated in bronchiectasis in particular in Asian populations [51, 52], whereas altered mycobiome was also found in bronchiectasis with increased abundance of *Aspergillus*, *Penicillium*, and *Cryptococcus* [53]. A recent seminal study by Mac Aogain et al. [54] delineated the integrated microbiomics in bronchiectasis by coprofiling bacteriome, mycobiome, and virome, and suggested that their mutual interactions were associated with key clinical features such as exacerbation frequency and antibiotic treatment. *Haemophilus* and *Pseudomonas* are also implicated in cystic fibrosis [55] and IPF [56, 57], with other pathogenic taxa such as *Staphylococcus* and *Stenotrophomonas* also commonly associated with both diseases [58, 59, 60, 61].

An important feature of the chronic respiratory diseases such as asthma and COPD are the inherent nature of heterogeneity, underpinned by different clinical phenotypes, inflammatory endotypes (the inflammatory pattern underlying a specific phenotype), and pathophysiology processes. Such heterogeneity has led to the proposal of a new paradigm for disease management not by disease “labels,” but according to “treatable traits” [62]. The lung microbiome differs substantially according to the specific phenotype or endotype of a disease, rendering difficulty in identifying disease‐specific microbiome features. In terms of clinical phenotype, the increase of Proteobacteria was most pronounced in a subgroup of COPD exacerbations with clinical evidence of bacterial infections, compared to the other exacerbation phenotypes such as those driven by viral infection or eosinophilic inflammation [42, 43]. In terms of inflammatory endotype, both neutrophilic inflammation and eosinophilic inflammation are evident in asthma and COPD with distinct airway microbiota. *Haemophilus* was predominant in neutrophilic inflammation, whereas certain less abundant taxa such as *Gemella*, *Granulicatella*, and *Campylobacter* were elevated in eosinophilic inflammation [63, 64, 65]. Differential mycobiome was also evident according to asthma endotypes, with *Fusarium*, *Cladosporium*, and *Aspergillus* specifically enriched in T2‐high asthma [66]. Our recent large‐scale integrative microbiome analysis on 1706 sputum samples from 510 patients has subdivided neutrophilic COPD into two subgroups based on the airway microbiome: the “*Haemophilus*‐predominant” and “balanced‐microbiome” subgroups. We found that these two subgroups have distinct inflammatory profiles, temporal variability, and microbiome–host interaction patterns, providing a novel framework for COPD “microbiome–host” cophenotyping [64]. Our recent study has further shown a differential airway resistome, a collection of antimicrobial‐resistant genes, in neutrophilic and eosinophilic COPD, suggesting the need to consider the inflammatory endotype for targeted antibiotic therapy [67].

### Acute lung diseases

Acute lung diseases are the pulmonary manifestation of an acute inflammation caused by local or systemic pathogenic infections such as pneumonia, sepsis, and the most recent COVID‐19. Acute lung injury (ALI) and the more severe ARDS are the primary syndromes for acute lung diseases in which lung microbiome is implicated. In a pioneer study by Dickson et al., [68] alteration of lung microbiota with enrichment of gut‐specific bacteria (i.e., *Bacteroides* spp.) was found in BAL samples of sepsis and ARDS patients, which was correlated with alveolar tumor necrosis factor‐α providing evidence for a potential role of gut–lung translocation in critically ill patients. In a follow‐up study by the same team on BAL samples of 91 critically ill patients, enrichment of gut‐specific taxa including *Lachnospiraceae* and *Enterobacteriaceae* was associated with poor clinical outcome including fewer ventilation‐free days and progression to ARDS [69]. Consistently, Panzer et al. [10] found that progression of critical ill patients to ARDS was associated with enrichment of *Enterobacteriaceae*, as well as taxa such as *Prevotella* and *Fusobacterium* that were related to smoking. By BAL sampling of 47 mechanically ventilated patients with or without ARDS, Kyo et al. [70] showed that *Staphylococcus*, *Streptococcus*, and *Enterobacteriaceae* were positively correlated with serum IL‐6 and mortality. Likewise, Kitsios et al. [71] found that enrichment of *Staphylococcus* and *Pseudomonadaceae* in tracheal aspirates was associated with worse clinical outcome of ventilated patients. Collectively, these results point to a consensus that airway dysbiosis with enrichment of gut‐related or other pathogenic taxa is characteristic of ALI/ARDS patients and is associated with poor clinical outcomes.

COVID‐19 has infected more than 500 million people worldwide and remains an ongoing global health crisis [72]. Acute infection of SARS‐CoV‐2 results in an uncontrolled inflammatory response and cytokine storm leading to ALI and ARDS [73, 74]. Respiratory dysbiosis could be a prominent feature of COVID‐19. By sampling the lower respiratory tract of critically ill patients with COVID‐19, Sulaiman et al. [75] found that poor clinical outcome was associated with lower airway enrichment with an oral commensal *Mycoplasma salivarium *and suggested that secondary bacterial infections may not drive mortality in COVID‐19. By a metatranscriptomic characterization of serial clinical specimens (sputum, nasal and throat swab, anal swab, and feces), Zhong et al., [76] identified *Burkholderia cepacia*, *Staphylococcus epidermidis*, and *Mycoplasma* spp. to be predominant in severely ill patients with codetection of other human respiratory viruses that were not identified in mildly affected patients suggesting the need to prevent the spread of antimicrobial resistance for hospitalized, severely ill COVID‐19 patients. Through a metatranscriptomic survey on 588 oropharyngeal swab specimens collected longitudinally from 192 COVID‐19 patients and 95 controls, Ren et al. [77] characterized the upper airway dysbiosis in COVID‐19 patients with a *Streptococcus*‐dominant microbiota specifically present in recovered patients. Specifically, *Streptococcus parasanguinis* in the upper airway could be a marker for the prognosis of non‐severe COVID‐19 patients.

### Other lung diseases

The lung microbiota is also implicated in other immune‐related lung diseases, including lung cancer, complications postlung transplantation, HIV, and tuberculosis. As chronic airway inflammation increases the susceptibility of lung cancer, the airway dysbiosis may be involved as a pathogenic mechanism [78]. In a pilot study, airway commensals *Veillonella* and *Megasphaera* were found to be enriched in BALF of patients with lung adenocarcinoma [79]. These findings are further supported by Huang et al., [80] who showed that the same two taxa were enriched in bronchial washing fluid in patients with lung adenocarcinoma versus squamous cell lung carcinoma. Studies have further associated the lung microbiome with key mutations and signaling pathways in lung cancer. Greathouse et al. [81] showed that increased lung *Acidovorax* was associated with the TP53 mutation. Tsay et al. [82] found that enrichment of oral taxa such as *Streptococcus* and *Veillonella* in the lower airways was associated with extracellular signal‐regulated kinase (ERK) and phosphatidylinositol 3‐kinase (PI3K) signaling. In a further mechanistic study, the same team showed that lung dysbiosis was associated with progression and poorer prognosis of lung cancer, and specifically, enriched oral taxa *Veillonella parvula* led to decreased survival, increased tumor burden, IL‐17 inflammation, and upregulated checkpoint inhibitor markers in a murine model of lung cancer [83]. Microbiome is associated with response to cancer immunotherapy [84]. Jang et al. [85] showed that *Veillonella dispar* was dominant in lung cancer patients with high PD‐L1 and responsive to immunotherapy, whereas *Neisseria perflava* was dominant in nonresponders, providing preliminary evidence for the implication of lung microbiome in lung cancer immunotherapy.

Lung transplantation is the last therapeutic option for patients with end‐stage lung disease. The most common complications postlung transplantation include acute and chronic lung allograft dysfunction. On analyzing BAL collected from 134 patients during 1‐year posttransplantation, Combs et al. [86] found that increased lung bacterial burden was predictive of chronic rejection and mortality, highlighting lung microbiome as a risk factor for lung allograft dysfunction. By combined amplicon sequencing and culture efforts, Das et al. [87] identified distinct “pneumotypes” in lung transplant recipients and established a link between microbiome, lung function, and clinical status post‐transplantation. Mechanistically, the same team further demonstrated that airway dysbiosis led to an imbalanced inflammatory and remodeling profiles of macrophages in the transplanted lung, which determined the airway immunologic tones [88]. In a multiomic study on BAL from lung donors and recipients, Watzenbock et al. [89] showed that the collective lung microbiome, metabolome, and lipidome are predictive of future lung function changes after transplantation.

Initiated by the Lung HIV Microbiome project, HIV is probably one disease area in which lung microbiome was first studied. One important early finding was the detection of *Tropheryma whipplei* in the lower airways of HIV patients, which was decreased after highly active antiretroviral therapy (HAART) [90]. Later studies showed increased *Prevotella* and *Veillonella* in HIV patients after 1 year of HAART treatment [91]. Mycobiome was also shown to be altered in HIV with the outgrowth of *Pneumocystis jirovecii* observed in both human and nonhuman primate models [92, 93]. For tuberculosis, *Mycobacterium tuberculosis*, its causative agent, was elevated in BAL of tuberculosis patients [94], although its detection rate by sequencing varies among studies [95]. Other taxa positively associated with tuberculosis include *Cupriavidus*, *Porphyromonas*, and *Streptococcus* [96, 97]. *Aspergillus* and *Candida* were also enriched in tuberculosis patients [97].

## MECHANISTIC INSIGHTS ON THE LUNG MICROBIOME

**Figure 3:**
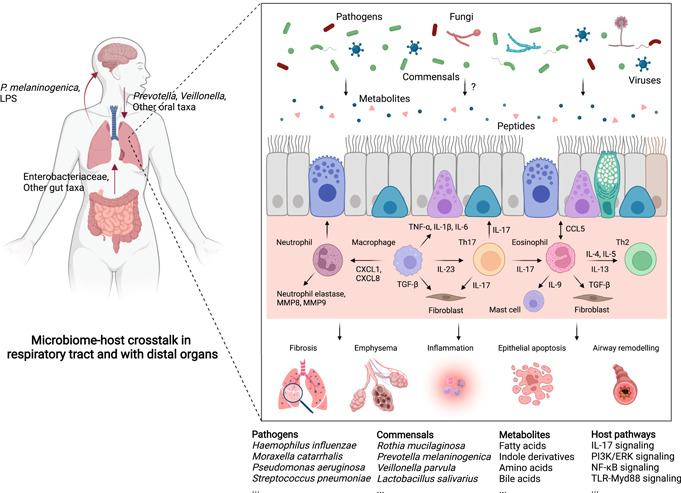
Microbiome–host crosstalk in the local respiratory tract and between the lung and distal organs. In the local respiratory tract, the pathogens or commensal bacteria, fungi, and viruses interact with each other and together interact with the host by producing or consuming metabolites or peptides, which are further involved in the molecular pathways underlying key pathophysiological processes such as fibrosis, emphysema, inflammation, epithelial apoptosis, and airway remodeling. The pathogens, commensals, their potential metabolites, and the host pathways modulated by microbiota based on evidence from mechanistic studies are shown below the pathway map. In between the lung and distal organs, enrichment of oral microbes (i.e., *Prevotella* and *Veillonella*) in the lower respiratory tract is shown to have complex effects on lung pathology. Enrichment of *Enterobacteriaceae* and other gut‐related taxa in the lower respiratory tract suggests the existence of a “gut–lung” axis. *Prevotella melaninogenica* and LPS from the lung microbiome are shown to regulate brain autoimmunity, implying a potential “lung–brain” axis. LPS, lipopolysaccharide.

The field of microbiome is rapidly advancing from correlations to causations between microbiome and diseases. Compared with the gut microbiome, whose mechanistic roles are increasingly well characterized, the effects and functions of the lung microbiome are only now beginning to be elucidated [98, 99]. Microbiome may contribute to chronic lung diseases through regulating host immunity and inflammation (Figure 3). By comparing germ‐free mice with special pathogen‐free mice with allergic airway inflammation, Herbst et al. [100] showed that the presence of commensal bacteria is critical for normal host immune function and control of allergic airway inflammation. By intranasally administering lipopolysaccharide (LPS) and elastase to establish a murine disease model that mimics key features of COPD, the same team further showed that the microbiota contributed to host IL‐17A inflammation and autoantibodies [101]. The lung microbiome also contributes to IPF progression. In a murine experiment by O'Dwyer et al., [102] lung dysbiosis was found to precede peak lung injury and persist afterwards. The microbiome's role in IPF was further examined in detail by Yang et al., [103] who showed that lung dysbiosis drove IL‐17B production and fibrosis through TLR‐Myd88 signaling. Inhaled corticosteroid (ICS) is a standard therapy for eosinophilic COPD patients, while it has the major risk of subsequent bacterial infection. By integrating human, cellular, and mouse data, Singanayagam et al. [104] showed that ICS suppressed a host defense protein named cathelicidin and resulted in airway dysbiosis with streptococci expansion, providing a mechanistic explanation for the risk of pneumonia after ICS use. While the effects of respiratory pathogens are mostly well established, the roles of commensal members of lung microbiota remain poorly understood. In a recent study by Rigauts et al., [105] *Rothia mucilaginosa*, a commensal member of airway microbiota, was found to alleviate airway inflammation by inhibiting the nuclear factor kappa B pathway. Other than inflammation, lung microbiota plays a role in regulating key host pathophysiological processes, such as oxidative stress, epithelial apoptosis, collagen deposition, mucus hypersecretion, and airway remodeling. D'Alessandro‐Gabazza et al. [106] found that a peptide corisin secreted by *Staphylocccus* induced lung epithelial apoptosis and collagen deposition toward acute exacerbations in IPF. Mouraux et al. [107] showed that airway microbiota was differentially related to airway anabolic or catabolic remodeling postlung transplantation, suggesting that the host–microbe interplay may determine remodeling activities in the transplanted lung. Lung microbiota is also essential in shaping host immune tolerance [108]. A hallmark study by Gollwitzer et al. [109] showed that lung microbiota promoted the development of T_regs_, leading to tolerance to allergens in neonatal mice via PD‐L1. The host responds to microbial colonization through the secretion of immunoglobulins (i.e., IgA, IgG, IgM), which is implicated in respiratory diseases. Collin et al. [110] reported increased IgA production in response to *Pseudomonas aeruginosa* infection in cystic fibrosis lung. Richmond et al. [111] reported that IgA deficiency in the airways resulted in persistent activation of innate immune response to lung microbiota, leading to a progressive COPD‐like phenotype.

The lung microbiome is also implicated in the crosstalk between the lung and distal organs (Figure 3). For example, multiple lines of evidence suggest that enrichment of oral taxa in the lower respiratory tract is a common phenomenon and exerts complex effects on lung pathology, by enhancing lung Th17 inflammation [112, 113], upregulating ERK and PI3K signaling [82], and increasing lung tumor burden [83]. The enrichment of *Enterobacteriaceae* and other gut‐related taxa in the lower respiratory tract during ALI suggests the existence of a “gut–lung” axis [114]. In support of the “gut–lung” axis, emerging evidence suggests the role of gut dysbiosis in respiratory diseases. Lai et al. [115] showed significantly altered gut microbiota in a COPD murine model and identified a commensal gut bacterium *Parabacteroides goldsteinii* that ameliorated COPD through LPS‐mediated antagonism of host TLR4 signaling. Li et al. [116] reported elevated lung inflammation, airway remodeling, and mucus hypersecretion in mice receiving fecal transplantation from COPD patients. Obese individuals have a higher risk of developing asthma, in which gut dysbiosis is also implicated. Michalovich et al. [117] showed that asthma severity was negatively associated with the fecal Akkermansia muciniphila level, and administration of this bacterium in an asthma murine model ameliorated airway hyperreactivity and inflammation. A recent ground‐breaking study by Hosang et al. [118] showed that lung dysbiosis with depletion of LPS‐enriched phyla increased the susceptibility of brain autoimmune diseases, proposing the first concept of a “lung–brain” axis.

## CURRENT CHALLENGES OF THE LUNG MICROBIOME

Notwithstanding these advances, the field of lung microbiome is still facing a myriad of challenges. First, as described previously, the low microbial biomass and excessive host contamination limit the application of metagenomics and metatranscriptomics that are fundamental to elucidating the microbiome functions. An efficient sample processing and sequencing procedure capable of capturing the airway metaomics with sufficient coverage and reasonable cost is required. Second, similar to other chronic diseases, most chronic lung diseases are heterogeneous, with different clinical manifestations, disease pathobiology, and airway microbiota. Disentangling the intricate relationships between microbiome and disease phenotypes and endotypes is a prerequisite to precisely define the microbiome's role in diseases. Third, microbiome produces metabolites or peptides that serve as ligands to interact with host receptors and trigger downstream signaling. Generally, little is known regarding the molecules specifically produced by the lung microbiome and their functions, as compared to those that are well characterized in the gut (i.e., short‐chain fatty acids, indole derivatives, amino acids, bile acids). A systems biology approach is required to generate an airway “microbial–host” multiomic landscape that delineates what airway microbes are capable of producing or consuming what molecules, and how these molecules interact with host proteins, pathways, and processes. Fourth, being able to precisely manipulate the airway microbiota in animal studies is crucial to assessing its functional impacts. Compared with techniques such as fecal microbiota transplantation, which is widely applied in gut microbiome studies, there is a lack of a standard procedure for manipulating the airway microbiota. Fifth, despite the power of next‐generation sequencing, being able to culture the microbes from the respiratory tract is instrumental for translational research. Although culturing respiratory pathogens is standard in clinical laboratories, little is known regarding the culturability of commensal lung microbiota. It is noteworthy that, using a culturomic strategy, Whelan et al. [119] showed that 82.1% of the operational taxonomic units in cystic fibrosis sputum were culturable.

## FUTURE AVENUES OF RESEARCH ON THE LUNG MICROBIOME

In light of these challenges, innovative experimental and analytical strategies tailored for the lung microbiome are paramount in moving the field forward. Longitudinal, interventional, and mechanistic studies are required to address causality. With these studies, it may be possible to answer more fundamental scientific questions in terms of the lung microbiome: What is the baseline status of healthy lung microbiome? How does lung microbiome respond to environmental stimuli? What are the roles of lung microbiome in different biological endotypes of respiratory diseases? How does lung microbiome differ in patients with different radiological features? Can microbiome be harnessed as a marker to facilitate the diagnosis, phenotyping, and prognosis of patients with respiratory diseases? What are the topographic structure and spatial dynamics of microbial communities in the lung? How do respiratory bacteria, fungi, and viruses interact with each other and how do they modulate host immunity? What are the key microbial metabolites that regulate host inflammation or other processes in the respiratory tract? What are the distal organs that can be influenced by lung microbiota and what are the mechanisms? Being able to answer these questions will eventually lead to a step closer toward our fundamental goal—to monitor the airway microbiome as a biomarker, and to manipulate the microbiome as a therapeutic target, toward precision medicine in respiratory and broader human diseases.

## AUTHOR CONTRIBUTIONS

Xinzhu Yi performed the literature review and wrote the manuscript. Jingyuan Gao performed the literature review. Zhang Wang supervized the project, and wrote and revised the manuscript. All authors have read the final manuscript and approved it for publication.

## CONFLICT OF INTEREST

The authors declare no conflict of interest.

## Data Availability

No new data and script were used in this paper. Supporting Information Materials (figures, tables, scripts, graphical abstract, slides, videos, Chinese translated version, and update materials) may be found in the online DOI or iMeta Science http://www.imeta.science/.
